# Supplementary material for: An improved method for culturing myotubes on laminins for the robust clustering of postsynaptic machinery
Source: Sci Rep. 2020 Mar 11;10:4524. doi: 10.1038/s41598-020-61347-x (PMC7066178; doi:10.1038/s41598-020-61347-x)

An improved method for culturing myotubes on laminins for the robust clustering of postsynaptic machinery

Marcin Pęziński,[1] Patrycja Daszczuk,[1] Bhola Shankar Pradhan,[1,2] Hanns Lochmüller,[3,4,5] and Tomasz J. Prószyński[1,2*]

[1]Laboratory of Synaptogenesis, Nencki Institute of Experimental Biology, Polish Academy of Sciences, Warsaw, Poland.

[2]Current address: Łukasiewicz Research Network – PORT Polish Center for Technology Development, Wrocław, Poland.

[3]Department of Neuropediatrics and Muscle Disorders, Faculty of Medicine, Medical Centre, University of Freiburg, Freiburg, Germany.

[4]Children's Hospital of Eastern Ontario Research Institute, University of Ottawa, Ottawa, Canada.

[5]Division of Neurology, Department of Medicine, The Ottawa Hospital, Ottawa, Canada.

*Correspondence:

Tomasz J. Prószyński

Łukasiewicz Research Network – PORT

Polish Center for Technology Development,

ul. Stabłowicka 147

54-066 Wrocław, Poland

Email: tomasz.proszynski@port.org.pl

Keywords: AChR clusters, neuromuscular, NMJ, postsynaptic machinery, synapse, laminin, developmental remodeling, human myotubes, podosomes

Supplementary Figure S1. Expression and clustering of AChR by cells grown on different substrata. (**a**) Myotubes grown on gelatin form small numbers of simple AChR clusters. (**b**) Example images of myotubes grown on indicated laminin combinations. (C) AChR-α1 protein levels in homogenates from C2C12 myotubes grown on different human laminins and gelatin. HEK293 cell lysates (HEK) was used as a negative control and tubulin was used as a loading control. Scale bars = 150 μm.

Supplementary Figure S2. Equal amounts of laminins were detected in the culturing wells after the coating procedure. (**a**) Control for laminin coating efficiency. After coating with each laminin, the wells were covered with sample buffer, and the collected material was resolved by SDS-PAGE and analyzed by silver staining. The results from three independent experiments are shown. (**b**) Predicted molecular weights for each laminin chain. (**c**) Myotube diameter of cells grown on different human laminin isoforms. (**d**) Average size of AChR clusters in C2C12 myotubes grown on each laminin isoform. $**p < 0.005$. For statistical analysis we used one way ANOVA with Dunnett test; laminin-111 used as a reference Error bars represent SEM values.

Supplementary Figure S3. Myoblast fusion index on different laminin isoforms. (a) Fusion index of C2C12 cells grown on different laminin isoforms. **(b)** Fusion index of human primary myoblasts grown on different laminin isoforms. **(c)** Representative images of C2C12 cells at the day 3 of fusion on indicated laminins. The cells were stained with DAPI to visualize nuclei (blue) and anti-myosin heavy chain antibody to label differentiated cells (green). $*p < 0.05$, $***p < 0.0005$. For statistical analysis we used one way ANOVA with Dunnett test; laminin-111 used as a reference. Scale bar = 100 μm.

Supplementary Figure S4. Rapsyn expression and localization in cells cultured on different laminin isoforms. (a) Rapsyn (red) localization to AChR clusters (green) in C2C12 myotubes. **(b)** Western blot analysis of rapsyn expression in homogenates of C2C12 cultures grown on different laminin isoforms. Tubulin was used as a loading control. Scale bar = 6 μm.

Supplementary Figure S5. Western blot analysis of AChR, tubulin, and rapsyn expression in C2C12 myotubes - scans of whole gels. (a) Myotubes express similar levels of AChR-α when grown on all tested laminin isoforms. Tubulin was used as loading control, both gels were processed at the same time and under the same conditions. **(b)** The same experiment as above, with lysate from myotubes grown on gelatin as an additional control. (c) Myotubes express similar levels of rapsyn when grown on all tested laminin isoforms. Tubulin was used as loading control, both gels were run at the same time.

Supplementary Figure 1.

a

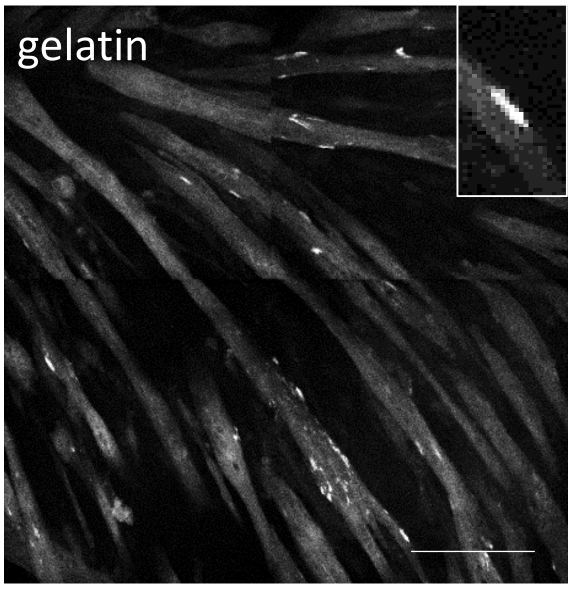

b

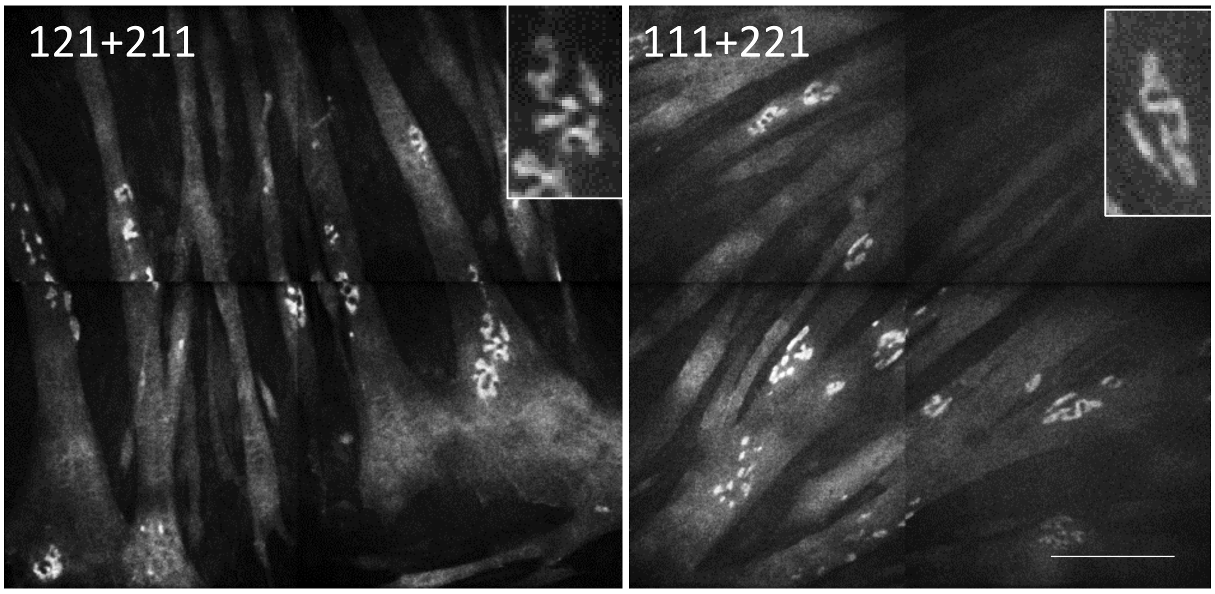

c

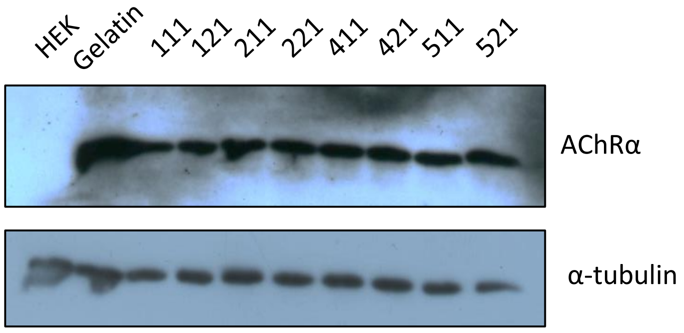

Supplementary Figure 2.

a

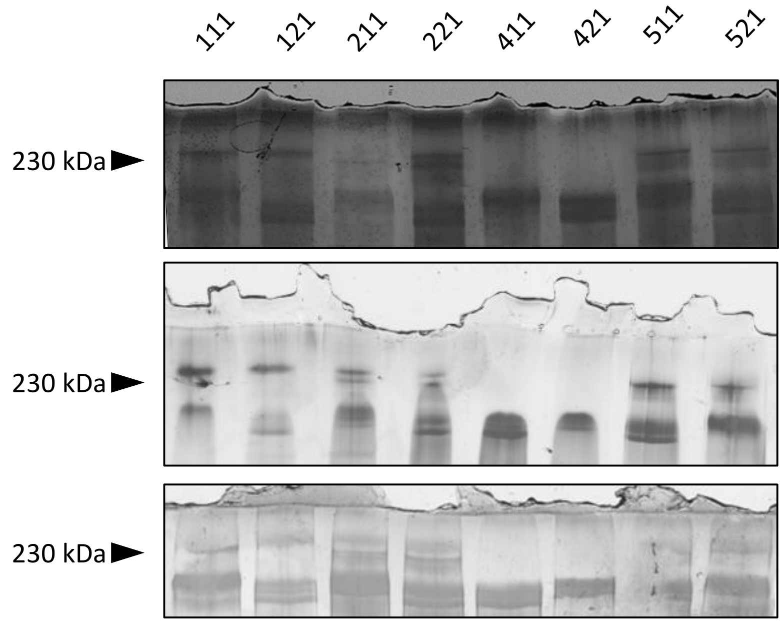

b

Laminin chain	MW (kDa)
α1	337
α2	343
α4	203
α5	400
β1	198
β2	196
γ1	178

c d

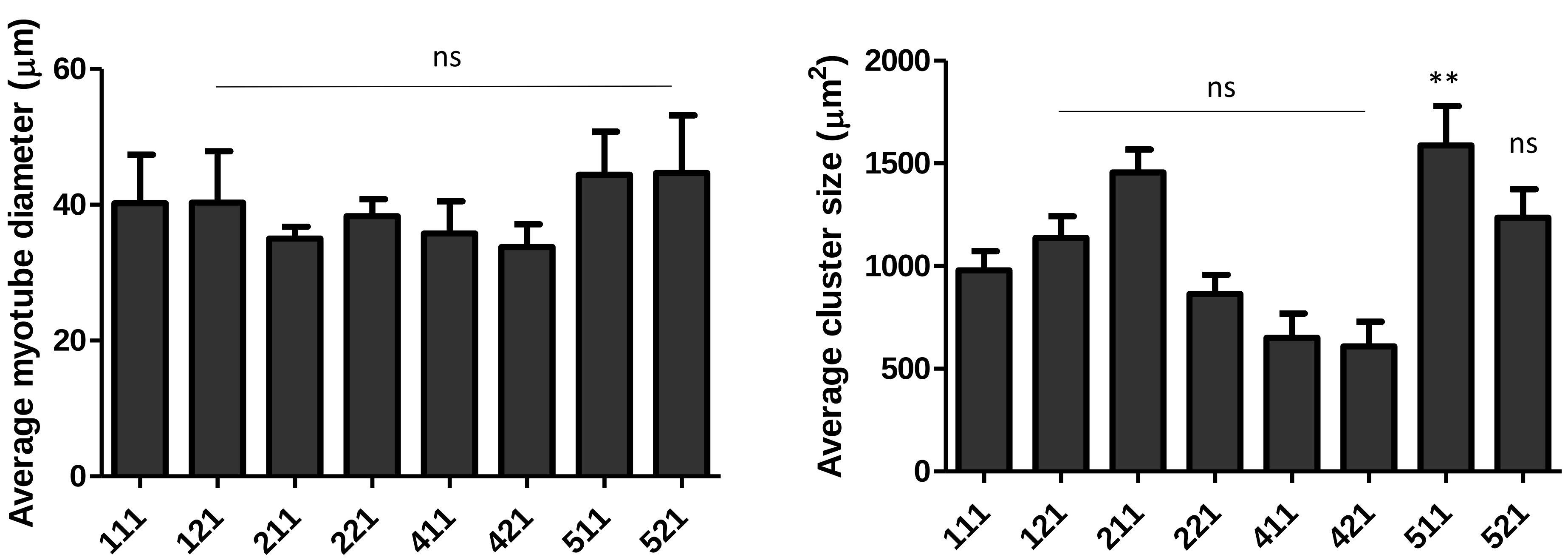

Supplementary Figure 3.

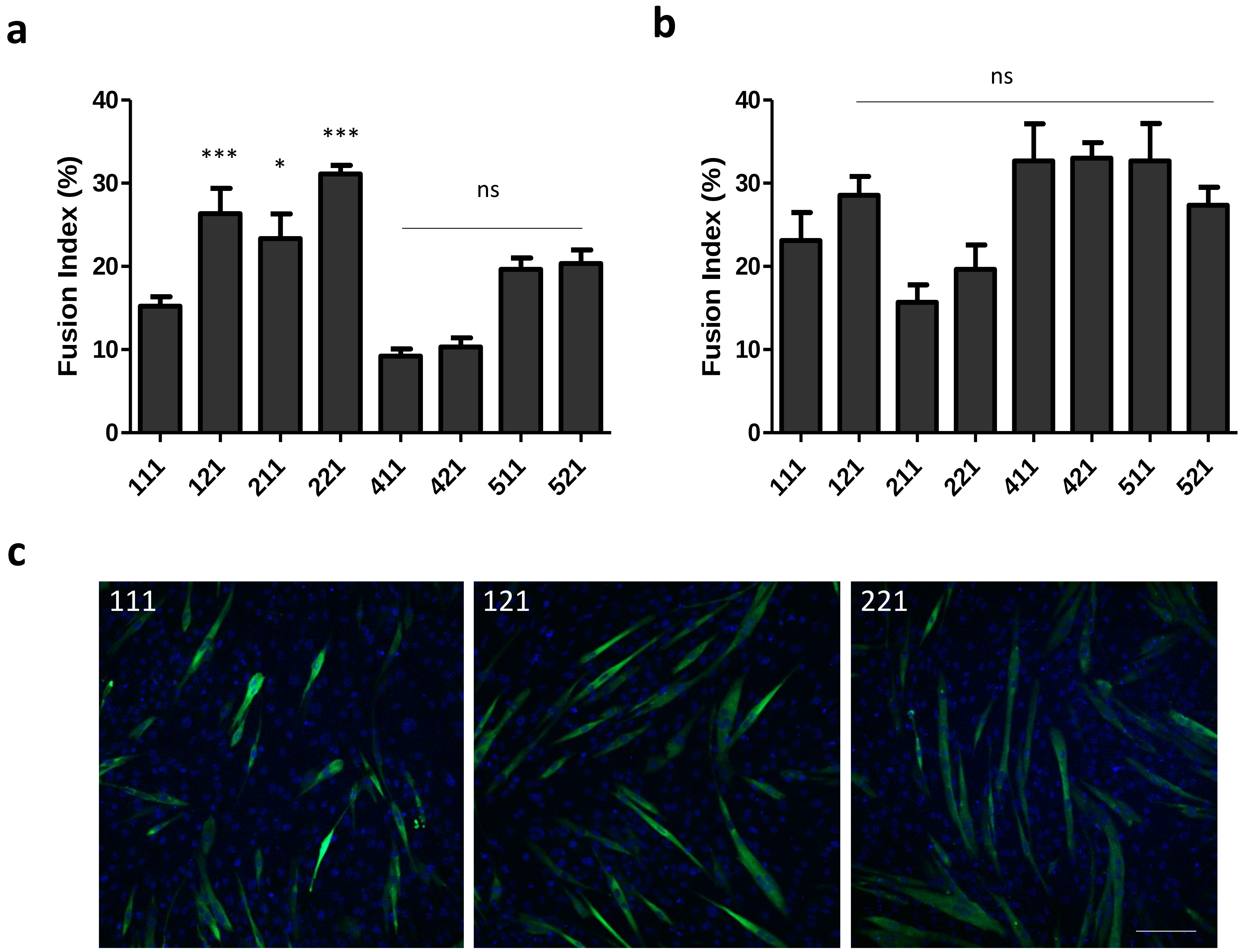

Supplementary figure 4.

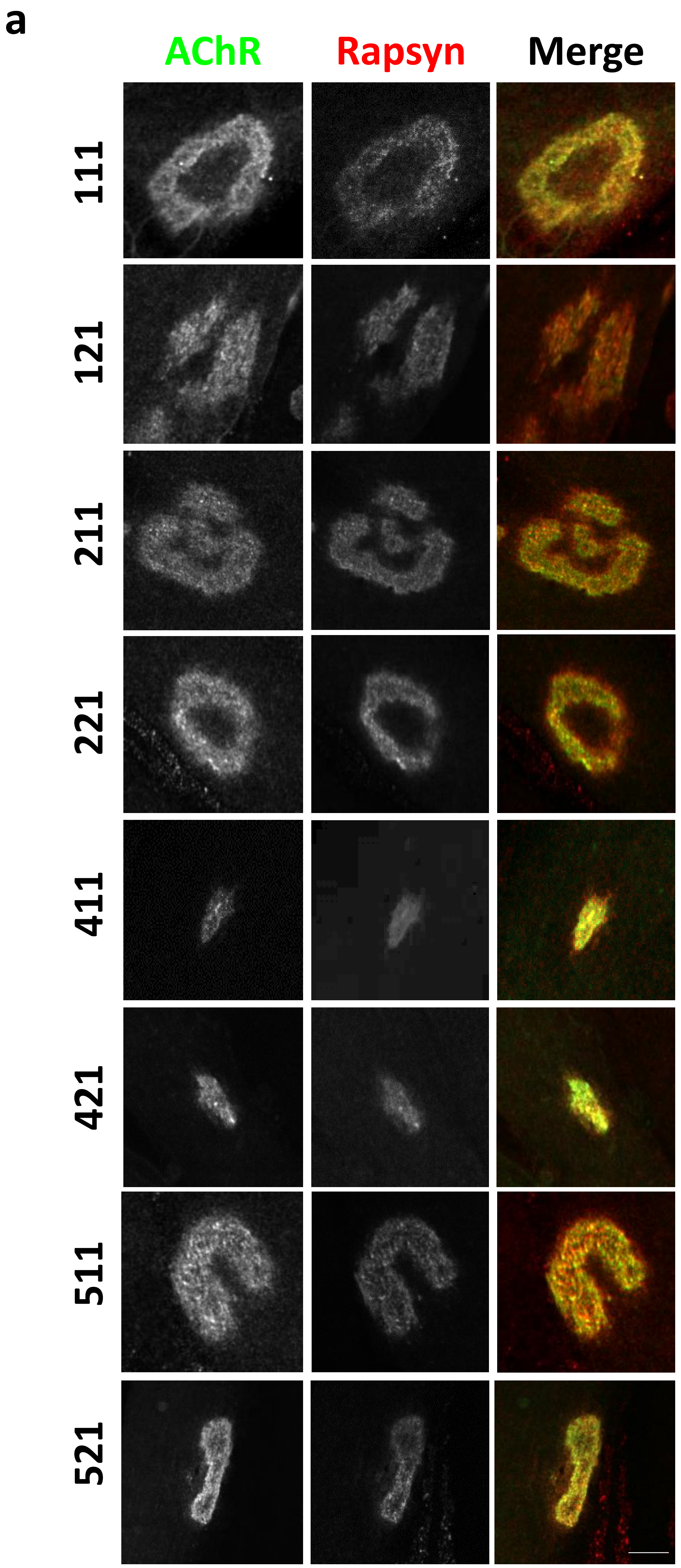

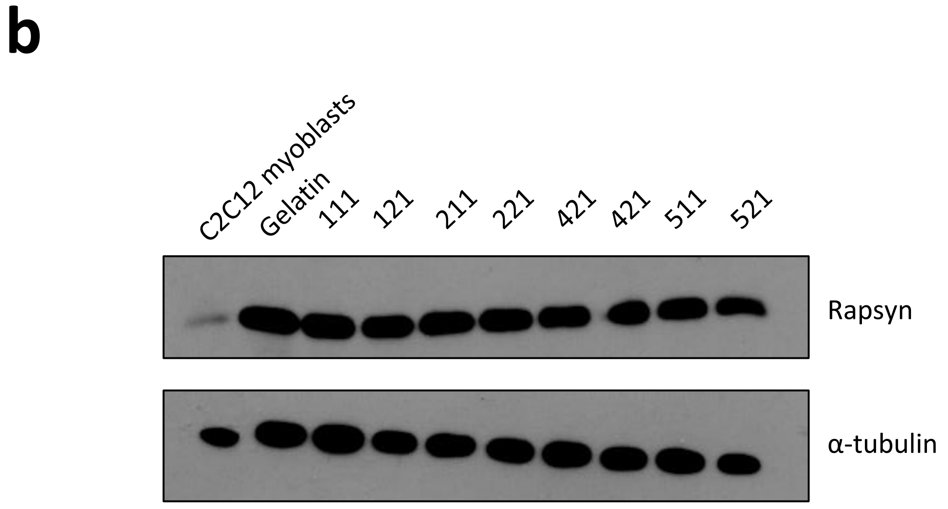

Supplementary figure 5.

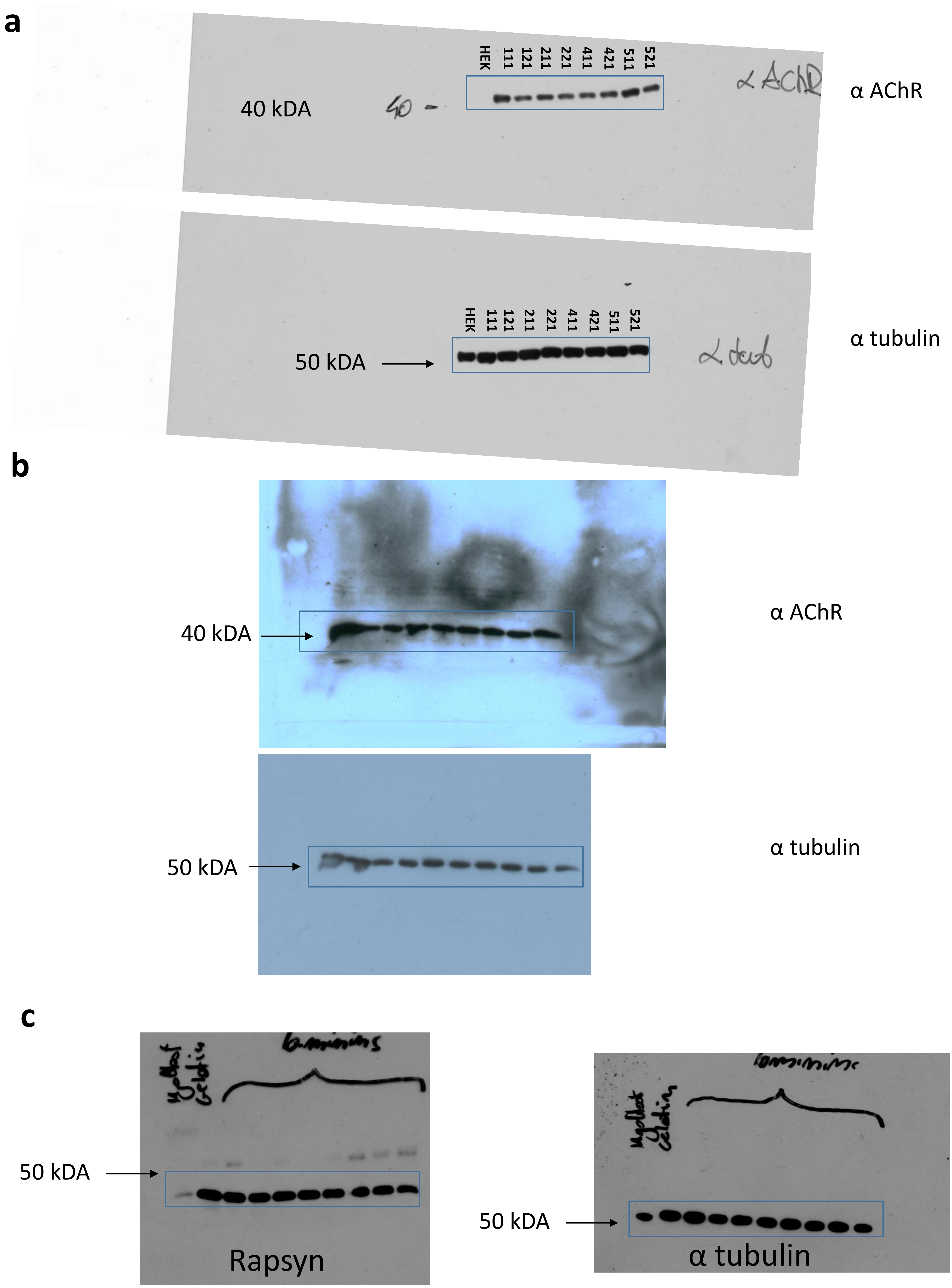

Supplement: Supplementary file 1 — Supplementary figures. [file 41598_2020_61347_MOESM1_ESM.pdf]
